# Microcornea and Thickened Lens in Angle Closure following Nonsurgical Treatment of Retinopathy of Prematurity

**DOI:** 10.1155/2020/7510903

**Published:** 2020-05-06

**Authors:** Ta C. Chang, Kimberly D. Tran, Linda A. Cernichiaro-Espinosa, Ella H. Leung, Alana L. Grajewski, Elizabeth A. Hodapp, Mohamed F. Abou Shousha, Audina M. Berrocal

**Affiliations:** ^1^Bascom Palmer Eye Institute, Miami, FL, USA; ^2^Sanford Eye Center, Sioux Falls, SD, USA; ^3^Asociación para Evitar la Ceguera en México, Mexico City, Mexico; ^4^Georgia Retina, Cartersville, GA, USA

## Abstract

**Purpose:**

To characterize the clinical features in young patients with angle closure and to determine the characteristics associated with acquired anterior segment abnormality following retinopathy of prematurity (ROP) treatment.

**Methods:**

We performed two retrospective case-control series. In the first series, we identified consecutive young angle closure patients without prior surgeries, with and without a history of ROP treatment; in the second series we identified consecutive patients who underwent ROP treatment, without and without anterior segment changes.

**Results:**

In the first series, 25 eyes of 14 consecutive angle closure patients were included: 19 eyes (11 patients, 78.6%) had a history of treated ROP, while 6 eyes (3 patients) belonged to full-term patients. The treated ROP eyes had significantly shallower anterior chambers (1.77 ± 0.17 mm vs 2.72 ± 0.18 mm, *P* < 0.0001) and thicker lenses (5.20 ± 0.54 mm vs 3.98 ± 0.20 mm, *P* = 0.0002) compared to the full-term controls. In the second series, 79 eyes of 40 patients were included, with median gestational age of 24.6 weeks. Acquired iridocorneal adhesion was noted in the eight eyes (10.1%) at a mean age of 4.7 years and was associated with prior zone 1 and plus disease (*P* = 0.0013), a history of initial intravitreal bevacizumab treatment (IVB, *P* = 0.0477) and a history of requiring additional IVB after initial treatment (*P* = 0.0337).

**Conclusions:**

Many young angle closure patients may have a history of treated ROP and may present with the triad of increased lens thickness, microcornea, and angle closure.

## 1. Introduction

Angle closure glaucoma in eyes without prior surgery is an uncommon finding in the pediatric population. In a tertiary referral center, excluding glaucoma following cataract surgery and glaucoma associated with systemic syndromes, glaucoma associated with nonacquired ocular anomalies accounts for less than 5% of patients with confirmed or suspected childhood glaucoma [1]. Etiologies of pediatric angle closure may include plateau iris, microspherophakia, iridociliary masses, anterior segment dysgenesis, a large lens, persistent fetal vasculature, chronic uveitis, and drug-induced [2–7]. Retinopathy of prematurity (ROP) has been associated with angle closure glaucoma, either as a sequela of chronic retinal detachment [8, 9] or as an acute process following panretinal photocoagulation (PRP). In this study, we present two retrospective case-control series. We first report a series of pediatric angle closure eyes with and without ROP in order to characterize the anatomic differences between these two groups. Then, we present a second series of infantile patients with a history of ROP treatment which we analyzed to determine the frequency and risk factors associated with acquired anterior segment abnormalities following ROP treatment.

## 2. Methods

We performed two retrospective reviews of medical records. Both protocols were approved by the Institutional Review Board of the University of Miami Miller School of Medicine, met the requirements of the Health Information Portability and Accountability Act, and adhered to the tenets of the Declaration of Helsinki. The eyes with prior incisional surgeries were excluded from both protocols.

### 2.1. First Series: Consecutive Young Angle Closure Patients

In the first series, we identified consecutive patients with angle closure who presented to our institution between 2014 and 2017. The patients with confirmed angle closure were included for analyses. Once enrolled, the patient's medical records were reviewed, and the following information was extracted: comorbidities, prior procedures, presence or absence of microcornea, qualitative description of lens morphology, biometry (if available), duration of follow-up, and management outcome.

### 2.2. Second Series: Consecutive Patients Who Underwent ROP Treatment

In the second series, consecutive patients who underwent ROP treatment at our institution between 2005 and 2014 with follow-up of at least 12 months were identified. The following information was extracted from the medical records: gestational age at birth, gender, laterality, date and type of initial ROP treatment, dates and types of subsequent ROP treatments, retinal disease category (zone 1 with plus disease, zone 1 with stage 3 disease, zone 2 with stage 2 and plus disease, vitreous hemorrhage, and unspecified), presence of iridocorneal adhesion, date of final visit, visual acuity, and IOP at the final visit. During much of the study period, the default treatment modality was PRP, as there were no data on the usage of intravitreal bevacizumab injection (IVB) in the management of ROP. IVB was performed as an initial treatment if there was sufficient media opacity to prevent adequate PRP and/or if the patient's systemic condition precluded PRP treatment. It was offered subsequently if the disease remained active despite full PRP, or if additional PRP was needed but considered not feasible due to the patient's condition.

### 2.3. Study Definitions

**Figure 1:**
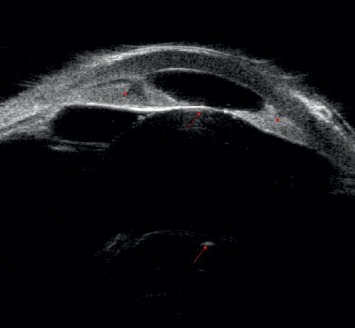
An example of pachyphakia based on qualitative anterior segment imaging. In this ultrasound biomicroscopy study, the lens morphology is nearly spherical, and the anteroposterior lens thickness is greater than 1.5 times the anterior chamber depth. Angle closure with iridocorneal adhesion is present. Arrows show anterior and posterior lens capsules; asterisks show iridocorneal adhesions.

Microcornea was defined as white-to-white corneal diameter (measured with either a caliper or a focused slit lamp light beam) less than 9.5 mm at any age, less than 10.5 mm in a child 1-2 years of age, and less than 11 mm in anyone 2 years of age or older. Angle closure was defined as present if there was less than 180° of visible trabecular meshwork or any peripheral anterior synechiae noted on gonioscopy, if iridocorneal apposition prior to dilation of 180° or greater was identified on anterior segment imaging, or if any visible iridocorneal adhesion of at least one clock hour was noted on direct observation of the anterior segment if gonioscopic or anterior segment imaging information was unavailable. Pachyphakia (Greek *pachý*(*s*) thick + Greek *phak*(*ós*) lentil (for lens)) was defined as lens thickness greater than 4.0 mm as measured by A-scan or estimated by anterior segment imaging (greater than approximately 2 standard deviations from the mean lens thickness) [1]. If the measurement was not available, anteroposterior lens thickness estimated to be greater than 1.5 times the anterior chamber depth on nonmydriatic slit lamp examination or on qualitative anterior segment imaging (Figure 1) was also defined as pachyphakia.

### 2.4. Statistical Analysis

A sensitivity analysis was done without the eyes that were missing the date of original treatment. Snellen acuities were converted to logarithm of minimum angle of resolution (logMAR) [2]. Fisher's exact tests were used to test for associations between the presence of an anterior segment abnormality with binary variables, and exact chi-square tests were used to test for these associations with categorical variables. Continuous variables were assessed for normal distributions. Independent sample *t*-tests were used to test for these associations for normally distributed continuous variables, and 2-sample Mann–Whitney–Wilcoxon tests were used to test for these associations for nonnormally distributed continuous variables. The failure event for the Cox proportional hazard survival analyses was defined by the presence of an acquired anterior segment abnormality. Logistic regression was used to determine whether years of follow-up was associated with an increased odds of failure. A *P* value <0.05 was considered statistically significant. All analyses were done using SAS version 9.4 (Cary, NC).

## 3. Results

### 3.1. First Series: Consecutive Young Angle Closure Patients

In the angle closure series, 25 eyes of 14 patients (9 females) were identified. Of these, 11 patients (9 female, 19 eyes) were premature with a history of treated ROP and presented at an average age of 11.9 ± 6.31 years (range 4.2 to 25.6 years). Three patients (2 females, 6 eyes) were full-term and presented at an average age of 11.9 ± 4.0 years (range 8.6 to 16.3 years). Three eyes of three patients with treated ROP were excluded due to prior incisional surgeries. Of the 19 eyes treated for ROP, 15 had undergone PRP, 2 had undergone combined PRP and IVB, and 2 had undergone cryotherapy. All of the treated ROP eyes had microcornea, while none of the eyes from full-term patients had microcornea. Of the 11 patients (19 eyes) with treated ROP, 7 patients (12 eyes) had thickened lenses characterized by imaging or biometric studies, while each of the remaining treated ROP eyes was noted to have a thickened or spherical lens without confirmatory ancillary testing. Of the three full-term patients (6 eyes), only one patient (2 eyes) had pachyphakia. Four of the treated ROP patients and all three of the full-term patients (8 and 6 eyes, respectively) had biometric measurements available which were performed at age 14.8 ± 7.43 and 13.8 ± 2.34 years, respectively (*P*=0.83). The treated ROP eyes had significantly shallower anterior chambers (1.77 ± 0.17 mm vs 2.72 ± 0.18 mm, *P* < 0.0001) and thicker lenses (5.20 ± 0.54 mm vs 3.98 ± 0.20 mm, *P*=0.0002) compared to the eyes of full-term patients. Axial lengths were not statistically different between the treated ROP vs full-term patients (20.6 ± 1.82 mm vs 22.0 ± 1.78 mm, respectively; *P*=0.1932). Results of the sensitivity analysis including only one eye per patient were similar to the results including all eyes (*P* < 0.0001 for anterior chamber depth difference, *P*=0.0129 for lens thickness difference). A similar proportion of treated ROP eyes and eyes of full-term patients required IOP-lowering medication (12/19 (63.2%) vs 4/6 (66.7%), respectively; *P*=0.86), while more eyes of full-term patients required surgery/laser (10/19 (52.6%) vs 4/6 (66.7%), respectively; *P*=0.040). As of the most recent visit, treated ROP eyes (mean age 13.5 ± 2.76 years) had poorer visual acuity (logMAR 0.77 ± 0.45 (approximately 20/118 Snellen equivalent) vs 0.19 ± 0.13 (approximately 20/31 Snellen equivalent), *P*=0.0053) and lower IOP (13.5 ± 2.76 mmHg vs 16.5 ± 3.27 mmHg, *P* < 0.0001) compared to eyes of full-term patients (mean age 16.5 ± 3.27 years), though this difference becomes insignificant on sensitivity analysis using only one eye in bilaterally affected patients (*P*=0.0551 and *P*=0.39 for logMAR visual acuity and IOP, respectively, Table 1).

**Figure 2:**
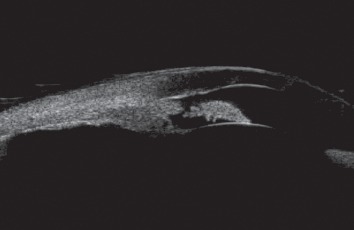
Ultrasound biomicroscopy examination of an eye that presented with acute angle closure and high pressure. Despite the presence of a patent peripheral iridotomy, the angle remained closed. The patient underwent treatment with lensectomy, intraocular lens implantation, and goniosynechialysis with satisfactory outcomes.

**Figure 3:**
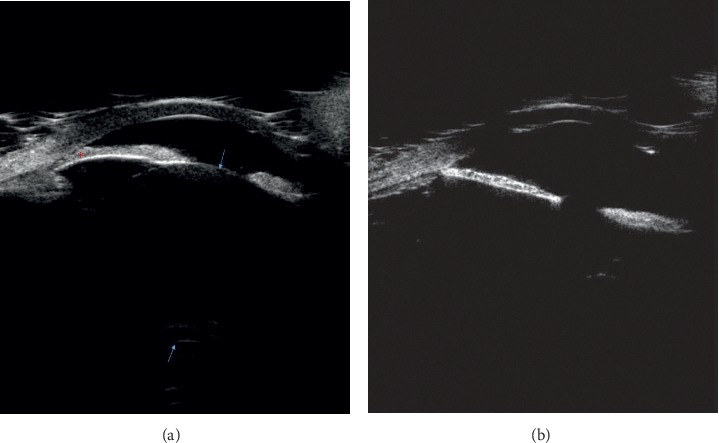
Ultrasound biomicroscopy examinations of the right and left eyes. Both eyes had a history of retinopathy of prematurity treated with panretinal photocoagulation. (a) The right eye was phakic, and the angle is closed. (b) The left eye was aphakic, and the angle is open. Arrows show anterior and posterior lens capsules; asterisks show iridocorneal adhesion.

One of the patients with a history of retinal ablation for ROP treatment presented with acute angle closure which failed to resolve despite the creation of laser peripheral iridotomies (Figure 2). She underwent lensectomy and goniosynechialysis sequentially in both eyes, and her IOP normalized without requiring medications. Another patient who presented for evaluation of angle closure had a phakic right eye with nearly complete iridocorneal apposition on ultrasound biomicroscopy and mildly elevated IOP. Her left eye, which was aphakic after lensectomy performed concurrent with retinal detachment surgery in infancy, had a mostly open angle (Figures 3(a) and 3(b)). The left eye was excluded from analysis.

### 3.2. Second Series: Consecutive Patients Who Underwent ROP Treatment

In the ROP infant series, 40 patients (79 eyes) with ROP treated with PRP and/or IVB were included; 50.0% were male, and 97.5% were treated bilaterally. The median gestational age (GA) at birth was 24.6 weeks (range 23–29 weeks). No eyes had iridocorneal adhesion at the time initial ROP treatment. The mean (standard deviation) age at which initial ROP treatment was performed (available in 33 of 40 patients) was 36.3 (2.87) weeks of gestation, with 63 of 79 eyes (79.8%) receiving PRP (mean of 1008 ± 512 laser spots) and 16 eyes (20.3%) receiving IVB. No eye received both PRP and IVB initially, and no bilaterally treated patients received discordant therapy between the right and the left eyes. The mean follow-up time for all eyes was 6.1 years.

Eight eyes (10.1%) of five patients (3 bilateral and 2 unilateral, with mean GA 23.9 ± 0.15 weeks at birth and mean follow-up time of 9.42 ± 3.38 years) had acquired iridocorneal adhesion noted at a mean age of 4.7 ± 3.14 years without retinal detachment or prior vitreoretinal/lens surgery. Three eyes (37.5%) developed elevated IOP that required IOP-lowering medical therapy, and one of the three eyes (12.5%) developed glaucoma [3]. The presence of iridocorneal adhesion (compared to its absence) was significantly associated with a past diagnosis of zone 1 and plus disease (25% vs 9.8%, *P*=0.0013), initial treatment with IVB (50% vs 16.5%, *P*=0.0477), and treatment with two or more IVB injections (25% vs 1.6%, *P*=0.0337; Table 2). In survival analysis, hazards ratio (HR) for the development of iridocorneal adhesion was significantly higher for eyes that required both PRP and IVB, additional IVB after initial treatment, and additional PRP after initial treatment. Results of the sensitivity analysis without the 14 eyes that were missing the date of original treatment were similar to the results with the complete dataset (Table 3).

## 4. Discussion

In our series of consecutive young patients with angle closure, 78.6% of patients had a history of ROP treated with retinal ablation. Microcornea and pachyphakia were noted in all angle closure eyes with treated ROP. This suggests that a history of retinal ablation for ROP may be associated with the triad of pachyphakia, microcornea, and angle closure (PMAC), and that patients with such a history may require close monitoring for the development of glaucoma. Increases in both lens thickness and corneal curvature in preterm children have been previously reported [4–6]. In a recent randomized study, infants who received PRP were significantly more likely than those treated with IVB to have very high myopia (≥8 diopters) [7]. This suggests that PRP may be associated with a higher magnitude of myopia compared to IVB by increasing lens thickness, axial length, or both. Based on this evidence, we hypothesize that infantile retinal ablation and heavy chorioretinal scarring may decrease the relative perfusion of the anterior segment, which could lead to the maldevelopment of the cornea and ciliary body ring and result in relative zonular laxity and pachyphakia. In one of our cases, IOP failed to normalize following the successful creation of a peripheral iridotomy, which supports the presence of a plateau iris-like configuration of the ciliary body resulting in angle closure [5]. While the mechanism by which retinal ablation results in ciliary body changes remains uncertain, the normalization of IOP following lensectomy suggests that lens-related ciliary body rotation may play a role in the pathogenesis of elevated IOP in PMAC, similar to the mechanism proposed for plateau iris syndrome [8].

After approximately 5 years of follow-up, nearly 10% of eyes in our infant cohort acquired iridocorneal adhesion. This suggests that even in successfully treated ROP, the anterior segment anatomy may continue to undergo pathologic changes over time. This finding is associated with past diagnosis of zone 1 and plus disease, initial treatment with IVB, and a history of requiring additional IVB after initial treatment. The mechanisms of acquired iridocorneal adhesion in type 1 prethreshold ROP treated with IVB are unclear. Based on the timing of our series, we believe that eyes that required IVB early in the treatment course likely had severe disease, with robust angiogenic drive that may have resulted in neovascularization of the angle and subsequent synechial angle closure. The majority of patients in our cohort (33 of 40) were treated prior to the first clinical trial of IVB in ROP treatment, [9] and at the time IVB was reserved for ROP cases in which media opacity precluded adequate PRP, or cases recalcitrant to conventional PRP. In this context, eyes amenable to initial PRP (as opposed to initial IVB) may have had less severe disease, as eyes that received initial PRP (compared to initial IVB) had a decreased risk of developing iridocorneal adhesion. In addition, eyes that required both PRP and IVB compared to single treatment alone had an increased risk of acquiring iridocorneal adhesion, while additional PRP after initial treatment yielded an increase in hazard of acquired iridocorneal adhesion comparable to additional IVB (HR 17.7 vs 21.25, respectively), suggesting that in our series, ROP disease severity, and not necessarily the use of IVB, may be linked to the risk of developing iridocorneal adhesion.

It is uncertain whether PMAC is a result of prematurity, ROP, retinal ablation, or a combination of these factors. In a recent randomized study, infants who received PRP were significantly more likely than those treated with IVB to have very high myopia (≥8 diopters) after 2.5 years [7]. This finding was supported by a 5-year retrospective case-control series, which noted significantly more myopia in PRP-treated infants compared to IVB-treated infants [10]. This suggests that while both treatment modalities resulted in myopia, PRP may be associated with a higher magnitude of myopia compared to IVB by increasing lens thickness, axial length, or both. It is even possible that there is a genetic basis for PMAC that also affects the likelihood of prematurity. Mutations in the *ADAMTS* family of genes have been implicated in both microcornea and lens morphology disorders [11–15], though it has not been associated with an increased risk of preterm birth. Similarly, maternal methylenetetrahydrofolate reductase gene *MTHFR* C677T polymorphism was associated with increased risks of both preterm birth and low-birth weight and MTHFR deficiency is implicated in a subtype of homocystinuria. However, there is currently no evidence linking *MTHFR* mutation to nonacquired anterior segment disorders. Thus, based on the available evidence, we hypothesize that while prematurity predisposes eyes to myopic refraction, infantile retinal ablation, rather than a genetic syndrome, leads to PMAC. Retinal ablation and heavy chorioretinal scarring may decrease the relative perfusion of the anterior segment, which could lead to the maldevelopment of the cornea and ciliary body ring and result in relative zonular laxity and pachyphakia.

There are several notable limitations to our study. First, we cannot discount the possibility of referral biases in both the ROP infant series and the angle closure series. Second, the actual incidence of iridocorneal adhesion in the ROP infant series may be underreported as subtle adhesion may not be apparent if the patient cannot cooperate with meticulous slit lamp examination. Third, our sample sizes for both series are modest. However, since angle closure glaucoma in eyes without prior surgery is an uncommon finding in the pediatric population, our series likely represents the largest of its kind. Lastly, even though our case series illustrated anterior segment abnormalities and angle closure associated with ROP, at this time we do not have direct evidence linking the acquired iridocorneal adhesions in the ROP infant series to the development of PMAC.

## 5. Conclusions

In summary, following nonsurgical ROP treatment, approximately 10% of patients may develop iridocorneal adhesions after 5 years. Many young patients with angle closure have a history of treated ROP, and half of them may require IOP-lowering therapy. Thus, a history of treated ROP accompanied with PMAC may warrant close monitoring for progressive anterior segment changes and/or referral to a pediatric glaucoma specialist for further evaluation. As the incidence of preterm birth increases and survival rate of preterm infants improves, anterior segment pathology in infants with treated ROP will likely become an increasingly important entity for pediatric ophthalmologists and anterior segment surgeons.

## Figures and Tables

**Table 1:** Baseline characteristics and management outcome of consecutive young angle closure patients with and without a history of treated retinopathy of prematurity (ROP).

	With treated ROP, *N* = 11, 19 eyes	Full-term patients, *N* = 3, 6 eyes	*P* value	Notes
Female, *N* (%)	9 (81.8%)	2 (66.7%)	0.50	
Age at presentation (years), mean ± SD^*∗*^	11.9 ± 6.31	11.9 ± 4.0	1.0	
Eyes with microcornea, *N* (%)	19 (100%)	0 (0%)	<0.0001	^*∗∗*^
Eyes with pachyphakia, *N* (%)	19 (100%)	2 (33.3%)	0.0012	^*∗∗*^
Biometry available	4 patients, 8 eyes	3 patients, 6 eyes		
Age at biometry (years), mean ± SD^*∗*^	14.8 ± 7.43	13.8 ± 2.34	0.83	
Anterior chamber depth (mm), mean ± SD^*∗*^	1.77 ± 0.17	2.72 ± 0.18	<0.0001	^*∗∗*^
Lens thickness (mm), mean ± SD^*∗*^	5.20 ± 0.54	3.98 ± 0.20	0.0002	^*∗∗*^
Eyes requiring IOP-lowering medications, *N* (%)	12 (63.2%)	4 (66.7%)	0.86	
Eyes requiring IOP-lowering laser/surgery, *N* (%)	10 (52.6%)	4 (66.7%)	0.04	^*∗∗*^
Age at most recent visit (years), mean ± SD^*∗*^	13.5 ± 2.76	16.5 ± 3.27	0.13	
Most recent logMAR^*∗*^ visual acuity, mean ± SD^*∗*^	0.77 ± 0.45	0.19 ± 0.13	0.0053^†^	^*∗∗*^
Most recent intraocular pressure (mmHg), mean ± SD^*∗*^	13.5 ± 2.76	16.5 ± 3.27	<0.0001^†^	^*∗∗*^

^*∗*^SD = standard deviation; logMAR = logarithm of minimum angle of resolution; ^†^this difference becomes insignificant on sensitivity analysis using only one eye in bilaterally affected patients (*P*=0.0551 and *P*=0.39 for logMAR visual acuity and IOP, respectively). ^*∗∗*^*P* value <0.05.

**Table 2:** Background characteristics of patients enrolled in the retinopathy of prematurity survey with and without acquired iridocorneal adhesion.

Characteristics	Iridocorneal adhesion absent, *N* = 71 eyes (*N* = 36 patients)	Iridocorneal adhesion present, *N* = 8 eyes (*N* = 4 patients)	*P* value	Notes
Gestational age, weeks (mean ± SD)^*∗*^	24.7 ± 1.35	23.9 ± 0.15	0.1912	
*Proportions*				
Male patients, *N* = 40	20 (50%)	20 (50%)	1.0	
Right eyes, *N* = 35	33 (46.5%)	2 (25.0%)	0.0726	In 9 eyes, the laterality was not specified
Patients with bilateral disease, *N* = 39	36 (100%)	3 (75%)	0.10	
Eyes with zone 1 and plus disease, *N* = 10	8 (11.3%)	2 (25%)	0.0014	^*∗∗*^
Eyes received intravitreal bevacizumab injection initially, *N* = 16	12 (16.9%)	4 (50%)	0.0488	^*∗∗*^
Eyes that required only one treatment, *N* = 55	54 (76.1%)	1 (12.5%)	0.0068	^*∗∗*^
Eyes that required additional laser only, *N* = 19	14 (19.7%)	5 (62.5%)		
Eyes that required both additional laser and bevacizumab injection, *N* = 5	3 (4.2%)	2 (25%)		

^*∗*^SD = standard deviation. ^*∗∗*^*P* value <0.05.

**Table 3:** Hazards ratio for the development of iridocorneal adhesion in the series of infants with treated retinopathy of prematurity.

Variables	Comparison	Hazards ratio (HR)	95% confidence interval		*P* value	Notes
			Low	High		
*Categorical*						
Gender	Male to female	1.13	0.17	7.29	0.8993	
Laterality	Right to left	1.50	0.67	3.34	0.3208	
Retinal disease category	Zone 1 and plus to others	1.50	0.29	7.60	0.6267	
	Zone 2, stage 2 with plus to others	0.31	0.04	2.18	0.2403	
	Vitreous hemorrhage to others	2.81	0.96	8.27	0.0605	
Initial treatment modality	PRP to IVB	0.12	0.02	0.84	0.0330	^*∗∗*^
Additional treatment modalities	PRP to none	12.64	1.30	122.50	0.0286	^*∗∗*^
	PRP + IVB to none	79.17	6.73	931.70	0.0005	^*∗∗*^
	PRP + IVB to PRP	6.26	0.42	92.86	0.1824	
	PRP to none or IVB	17.07	1.97	148.10	0.0100	^*∗∗*^
	IVB to none or PRP	21.25	1.54	293.60	0.0225	^*∗∗*^

*Continuous*	Unit					
Gestational age	1 week	0.58	0.26	1.28	0.1757	
Number of PRP spots	250 spots	0.47	0.20	1.10	0.0810	
Number of injections	1 injection	3.47	1.46	8.24	0.0049	^*∗∗*^

HR = hazards ratio; PRP = panretinal photocoagulation; IVB = intravitreal bevacizumab injection. ^*∗∗*^*P* value <0.05.

## Data Availability

This report contains all the available data from this study.
